# Management of Dynapenia, Sarcopenia, and Frailty: The Role of Physical Exercise

**DOI:** 10.1155/2020/8186769

**Published:** 2020-01-31

**Authors:** Ricardo Aurélio Carvalho Sampaio, Priscila Yukari Sewo Sampaio, Marco Carlos Uchida, Hidenori Arai

**Affiliations:** ^1^University of Campinas, Campinas, Brazil; ^2^Federal University of Sergipe, Lagarto, Brazil; ^3^National Center for Geriatrics and Gerontology, Obu, Japan

Aging is a result of physiological changes and their interactions with personal lifestyles, genetics, and chronic diseases. The musculoskeletal system and physical capabilities might decline with aging, and the transition from plateau to decline is determined by biological timing and individual life trajectories.

Dynapenia is defined as the loss of muscle strength that is not caused by neurologic or muscular diseases; it is a state in which reduced muscle strength is not necessarily accompanied by decreased skeletal muscle mass [1]. Indeed, muscle strength is presently the most reliable measure of muscle function, and when low muscle strength is verified, sarcopenia is imminent [2].

The recent European Working Group on Sarcopenia in Older People 2 (EWGSOP2) update recognized sarcopenia as a progressive and generalized skeletal muscle disorder diagnosed mainly by the presence of low muscle strength, and this low muscle strength overtook the role of low muscle mass as a primary parameter of sarcopenia [2]. Both dynapenia and sarcopenia reflect directly on older adults' activities of daily living, poor quality of life, falls and fear of falling, and the frailty syndrome and can lead to disability and mortality in older adults.

Given that physical exercise is an important nonpharmacological approach to promote healthy aging, the purpose of this special issue is to present applied studies that discussed the interaction among dynapenia, sarcopenia, frailty, and physical exercise in the context of aging. In addition, considering that many aspects of these conditions are better understood with all accumulated information during last years, it is expected that the articles published in this special issue will contribute adding high-quality information to this field of knowledge. A brief summary of all accepted papers is provided as follows.

In the paper by R. G. B. de Mello et al., a systematic review was conducted to identify randomized clinical trials (RCTs) which tested the effects of physical exercise programs to manage sarcopenia components in older individuals. The authors found 301 studies for inclusion, and after screening, five RCTs were included. All trials tested the efficacy of isolated exercise programs to improve sarcopenia components in older adults compared with no physical intervention. Resistance training was the main intervention component in all included trials compared with inactive control groups (mainly health education). They concluded that resistance training protocols can improve muscle strength and physical performance in older people previously diagnosed with sarcopenia compared with inactive control groups.

The paper by A. G. de Resende-Neto et al. presented a randomized crossover trial that analyzed the efficacy of functional training and traditional training (i.e., machine-based) in body composition and determinants of physical fitness in Brazilian older women. Forty-eight participants performed two 12-week periods of training. The authors verified that both functional and traditional training promoted improvements in physical fitness for daily activities in older women and may be prescribed in combination with optimizing general fitness levels in older adults.

Regarding the work by J. A. Vaccaro et al., a focused review was carried out to identify and integrate the evidence and lack of strategies to prevent frailty in older adults with diabetes. The authors found some evidence that motivational approaches have worked for older adults with various chronic disease conditions. However, studies applying motivational strategies are lacking for frail older adults with type 2 diabetes. Then, interventional studies specifically for this population are needed for the success in promoting health behavior changes to reduce frailty.

The paper by O. J. Perkin et al. presented a pilot study that examined the effect of a 28-day unsupervised home-based exercise intervention on indices of leg strength and muscle size in healthy older adults. Twenty participants were randomly assigned to either maintain their habitual physical activity levels (i.e., control) or undertake “exercise snacks” (ES) that included five leg exercises twice daily. Improvements of ES in leg muscle function and size in older adults were found when comparing with the control group.

In addition, the paper by M. Tomioka et al. presented a 12-month participation in and impact of Enhance®Fitness (i.e., a low-cost group exercise program designed specifically for older adults) on physical performance among older adults in Hawaii. This study analyzed several physical function tests at baseline and at 4, 8, and 12  months. Moreover, the authors compared the characteristics of participants who were engaged in all research activities with those who dropped out in order to gain insights on participant's adherence to exercise. Of all 1,202 older adults with baseline data, 427 (35.5%) were continuously enrolled in the program for 12  months. Participants' physical performance measures improved after 4  months, continued to improve until 8  months, and were maintained thereafter. Common reasons for dropping out (*n* = 775) were illness, relocation, time conflicts, lost interest, and transportation issues.

Finally, the paper by M. M. A. Mesquita et al. aimed to analyze the reproducibility of a protocol using the maximal isometric strength test of the trunk in older women. The rationale of this study was that changes related to trunk muscles, due to their important role in performing activities of daily living and in terms of better functional performance, is an important parameter to evaluate the state of health of an individual. In addition, the authors supported the development of alternative reliable and low-cost tests and protocols for evaluating muscle strength in older people.
